# Polarization‐Dependent Sum‐Frequency‐Generation Spectroscopy for In Situ Tracking of Nanoparticle Morphology

**DOI:** 10.1002/anie.202300230

**Published:** 2023-04-04

**Authors:** Verena Pramhaas, Holger Unterhalt, Hans‐Joachim Freund, Günther Rupprechter

**Affiliations:** ^1^ Institute of Materials Chemistry TU Wien Getreidemarkt 9/BC 1060 Vienna Austria; ^2^ Fritz-Haber-Institut der Max-Planck-Gesellschaft Faradayweg 4–6 14196 Berlin Germany; ^3^ Current address: ZKW Lichtsysteme Scheibbser Strassse 17 3250 Wieselburg Austria; ^4^ Current address: Robert Bosch GmbH Tübinger Straße 123 72762 Reutlingen Germany

**Keywords:** Metal Nanoparticles, Particle Morphology, Sum Frequency Generation, Vibrational Spectroscopy, in Situ Spectroscopy

## Abstract

The surface structure of oxide‐supported metal nanoparticles can be determined via characteristic vibrations of adsorbed probe molecules such as CO. Usually, spectroscopic studies focus on peak position and intensity, which are related to binding geometries and number of adsorption sites, respectively. Employing two differently prepared model catalysts, it is demonstrated that polarization‐dependent sum‐frequency‐generation (SFG) spectroscopy reveals the average surface structure and shape of the nanoparticles. SFG results for different particle sizes and morphologies are compared to direct real‐space structure analysis by TEM and STM. The described feature of SFG could be used to monitor particle restructuring in situ and may be a valuable tool for operando catalysis.

## Graphical abstract

Polarization‐dependent sum‐frequency‐generation (SFG) spectroscopy of adsorbed CO was employed to characterize the morphology of oxide‐supported Pt and Pd nanoparticles. Via the *I*_ssp_/*I*_ppp_ ratio, the orientation of CO relative to the macroscopic surface normal reveals the abundance of inclined/curved facets.

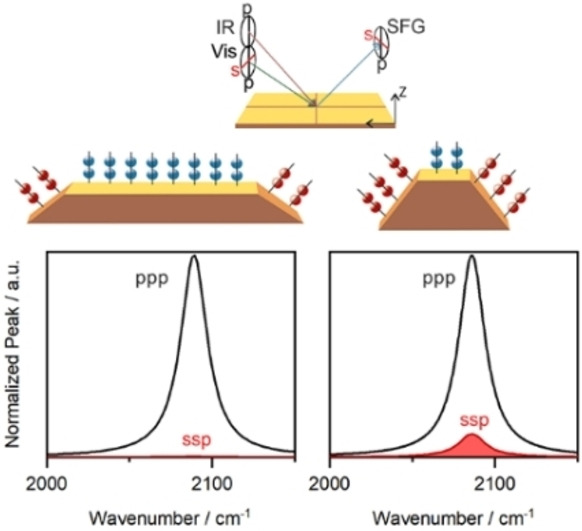

Sum frequency generation (SFG) is a powerful non‐linear optical vibrational spectroscopy. Upon simultaneous excitation by a broadband or wavelength‐scanned mid‐infrared (IR) and a fixed narrowband visible (VIS) laser pulse, a signal is generated at the sum of the incident frequencies. The underlying process is limited to non‐centrosymmetric media (e.g., surfaces/interfaces) and exhibits inherent surface‐sensitivity.[^1^, ^2^] Thus, different from conventional IR spectroscopy, SFG *only* measures vibrations of surface adsorbed molecules, even when the same molecules are also present in, e.g., a gas phase[^3^, ^4^, ^5^, ^6^, ^7^] (for differentiating surface and gas phase contributions IR absorption spectroscopy requires polarization‐modulation^[8]^). Accordingly, SFG can also characterize molecules at air‐liquid and liquid‐liquid interfaces and even examine “buried” interfaces inside solids.[^9^, ^10^, ^11^, ^12^] Furthermore, the strong dependence of the coherent SFG light on the ordering and abundance of the probed bonds enables structure/coverage evaluation, while using different IR and vis polarization combinations allows identification of bond orientation,[^13^, ^14^, ^15^] due to the tensor character of the second order non‐linear susceptibility. SFG has been applied to studies of interface phenomena in many fields, including electrochemistry,^[16]^ photocatalysis,^[17]^ plasmonics,^[18]^ polymers,^[19]^ self‐assembly,^[20]^ and nanomedicine.[^21^, ^22^]

Herein, we applied polarization‐dependent SFG to examine molecular adsorption on oxide supported metal nanoparticles (NPs) of Pt and Pd. Given that the used probe molecule CO adsorbs in the same orientation to top and side facets of Pt and Pd surfaces,[^13^, ^14^, ^15^, ^23^] the intensity ratio of different polarization combinations in SFG measurements is affected by the shape of the NPs rather than by varying adsorption tilt angles. We demonstrate that the spectral intensity ratio mirrors the shape of different NPs in two different model catalyst systems, matching the direct morphology characterization by transmission electron microscopy (TEM) and scanning tunneling microscopy (STM). This enables the use of SFG for in situ evaluation of average NP morphology for reactions that involve CO or are not negatively affected by CO probe molecules.^[24]^

The basics of sum frequency generation spectroscopy have been described in the literature,[^1^, ^2^, ^6^, ^8^, ^10^, ^11^, ^12^, ^13^] with the SFG intensity I_SFG_ depending linearly on the intensity of the incident beams, I_IR_ and I_VIS_, and the absolute square of the second order nonlinear susceptibility *χ* ^(2)^. A scheme of the beam propagation and the possible beam polarization directions is displayed in Figure 1a. It has been shown that on metals only two polarization combinations, i.e., ppp and ssp, yield a significant SFG signal:[^25^, ^26^]

(1) $$\chi_{eff,ppp}^{\left(2\right)}=\;-L_{xx}\left(\omega_{SFG}\right)L_{xx}\left(\omega_{Vis}\right)L_{zz}\left(\omega_{IR}\right)\cos\left(\alpha_{SFG}\right)\cos\left(\alpha_{Vis}\right)\sin\left(\alpha_{IR}\right)\chi_{xxz}-L_{xx}\left(\omega_{SFG}\right)L_{zz}\left(\omega_{Vis}\right)L_{xx}\left(\omega_{IR}\right)\cos\left(\alpha_{SFG}\right)\sin\left(\alpha_{Vis}\right)\cos\left(\alpha_{IR}\right)\chi_{xzx}+L_{zz}\left(\omega_{SFG}\right)L_{xx}\left(\omega_{Vis}\right)L_{xx}\left(\omega_{IR}\right)\sin\left(\alpha_{SFG}\right)\cos\left(\alpha_{Vis}\right)\cos\left(\alpha_{IR}\right)\chi_{zxx}+L_{zz}\left(\omega_{SFG}\right)L_{zz}\left(\omega_{Vis}\right)L_{zz}\left(\omega_{IR}\right)\sin\left(\alpha_{SFG}\right)\sin\left(\alpha_{Vis}\right)\sin\left(\alpha_{IR}\right)\chi_{zzz}$$

(2) $$\chi_{eff,ssp}^{\left(2\right)}=L_{yy}\left(\omega_{SFG}\right)L_{yy}\left(\omega_{Vis}\right)L_{zz}\left(\omega_{IR}\right)\sin\left(\alpha_{IR}\right)\chi_{yyz}$$

**Figure 1:**
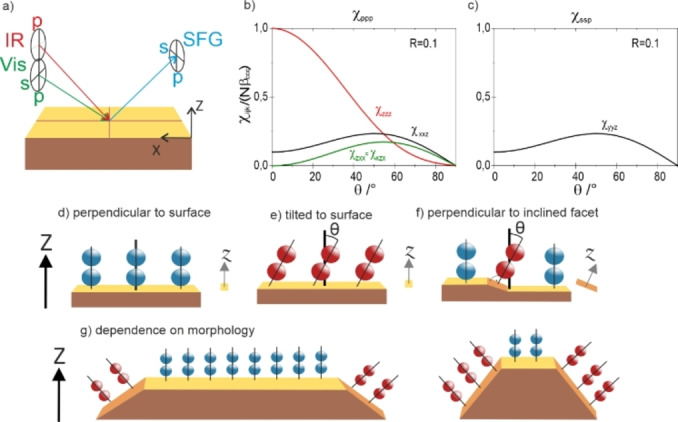
a) Possible polarizations of SFG beams and their orientation in the laboratory frame. b), c) simulated contributions to the effective nonlinear susceptibility by the non‐vanishing elements ppp and ssp polarization combination, using a low R=β_aac_/β_ccc_ of 0.1. d)–g) influence of inclined surface facets on the bond orientation of CO relative to the macroscopic surface normal.

*L*_ab_ denotes the Fresnel factors and *χ_ijk_* denotes the non‐vanishing tensor elements as given in the Supporting Information. *χ*_ppp_ is a linear combination of several elements (Figure 1b), weighted by the Fresnel factors. For the current measurement geometry, it has a maximum at 0° bond tilt angle and monotonously decreases with increasing tilt (with *χ_zzz_* as main contribution).^[13]^ In comparison, *χ*_ssp_ is directly proportional to *χ_yyz_*, so the ssp intensity increases with increasing tilt angle *θ* between the linear CO molecule and the surface normal Z, reaching a maximum around 45 to 50° (see Figure 1c), depending mainly on the molecular tensor element ratio R=β_aac_/β_ccc_.[^13^, ^14^, ^15^]

Therefore, a comparison of *I*_ppp_ and *I*_ssp_ allows to monitor the tilt angle of molecular bonds, but most SFG studies of metal‐gas interfaces typically reported only the ppp spectra, as these yield the strongest signal. A key aspect of the observations reported herein is schematically illustrated in Figure 1d–f: molecules perpendicularly adsorbed (blue) on a planar surface have no tilt to either the local surface normal *z* or the macroscopic normal *Z* (Figure 1d), while molecules adsorbed in a tilted geometry (red) on a flat surface are tilted with respect to both *z* and *Z* (Figure 1e). As SFG is a macroscopic technique, even a molecule that is adsorbed perpendicularly on an *inclined* facet (parallel to *z*) is tilted from the macroscopic surface normal *Z* (Figure 1f). Therefore, it contributes to the signal like a molecule with tilted adsorption geometry. Variations in the particle morphology as illustrated in Figure 1g thus lead to different intensity ratios for SFG measurements of different polarization combinations, which reflect the NP shape.

In this study, two supported metal NP systems were investigated, Pt on ZrO_2_ and Pd on Al_2_O_3_. Measurements were performed in two comparable setups, each consisting of an ultra‐high vacuum (UHV) surface‐preparation and analysis chamber and an attached SFG spectroscopy cell for in situ experiments from UHV to atmospheric pressure, which have been described in detail.[^27^, ^28^] As the metal nanoparticles were >3.5 nm, no support effects are to be expected.[^29^, ^30^]

The Pt/ZrO_2_ model catalysts consisted of a 42 nm thick zirconia film—grown by 400 cycles of atomic layer deposition (ALD) on a Si(100) wafer—and Pt deposits prepared by different numbers of ALD cycles (10 to 250).^[33]^ TEM images showed that using 10 Pt cycles produced Pt particles roughly 6 nm in size, whereas 250 deposition cycles formed a homogeneous Pt film of uniform thickness of approximately 10 nm (Figure 2). The latter serve as a bridge between previous single crystal studies and the current NP results. For characterization by electron microscopy, X‐ray diffraction and photoelectron spectroscopy refer to reference [33].^[33]^

**Figure 2:**
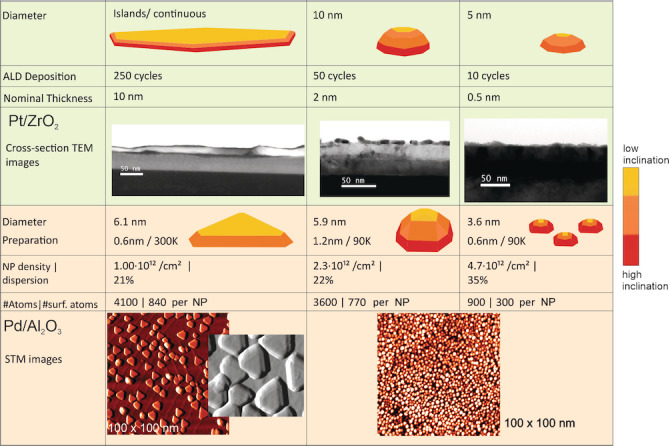
Morphology of supported Pt and Pd nanoparticles. The ALD‐deposited Pt particles initially grow with pyramidal shape that becomes more rounded upon increasing the Pt amount. Highest Pt exposure leads to coalescence, forming smooth islands and finally continuous smooth films. The Pd particle size is varied by different substrate temperature and Pd amount during PVD. At 300 K, particles grow with truncated cuboctahedral shape exhibiting a large flat (111) top facet. At 90 K, rougher, half‐spherical NPs are formed, also with higher nucleation/particle density. STM images adapted from with permission from Refs. [31] and [32].

The second set of samples, well‐defined Pd NPs, were grown by physical vapor deposition (PVD) on alumina thin films in UHV.[^31^, ^32^, ^34^, ^35^, ^36^] Figure 2 shows STM images of Pd NPs grown on Al_2_O_3_/NiAl(110) at 300 K and 90 K substrate temperature. Pd NPs grown at 300 K are truncated cuboctahedra with distinct smooth (111) and (100) facets, whereas particles grown at 90 K appear rather rounded/irregular (i.e., the facets are rougher with more steps/defects). For the samples discussed below, a nominal thickness of 0.6 nm Pd was deposited at 300 and 90 K, yielding well‐facetted Pd NPs of 6.1 mean size and rougher Pd NPs of 3.6 nm mean size, respectively. NPs of 5.9 nm mean size were grown at 90 K by depositing a nominal thickness of 1.2 nm Pd (using spot profile analysis of low energy electron diffraction (SPA‐LEED) for size/NP density analysis^[35]^). Similar to the Pt samples, these larger particles are higher than particles grown with less Pd and thus have more pronounced sideward facing facets. Additional information can be found in the Supporting Information.

For both model catalyst systems, polarization‐dependent SFG measurements of adsorbed CO molecules revealed three important variables: The first is the peak position, which provides information on the local adsorption geometry (on‐top/bridge/hollow). The exact resonance position, especially for on‐top CO, is further affected by the surface roughness (metal coordination number) and the CO coverage (via dipole‐dipole coupling and chemical shift).^[37]^ The second is intensity, which depends on the abundance of adsorption sites, coverage, ordering and vibrational coupling of adsorbed CO.[^7^, ^8^, ^37^] SFG measurements for extracting the size of Pt^[38]^ and Pd particles^[39]^ using only ppp polarization, have been reported previously. In these studies, particle structure was identified by comparing the peak positions and intensities and using different theoretical simulation models as references. The third measured variable is the *I*_ssp_ to *I*_ppp_ ratio, which provides information on the average orientation of the C−O bond with respect to the *macroscopic* surface normal. Polarization dependent (ssp and ppp) SFG spectra of CO adsorbed on Pt and Pd single crystals have been reported[^8^, ^13^, ^14^, ^15^, ^40^] and are used for benchmarking our results.

Figure 3 shows the ppp (black) and ssp (red) spectra of the ALD Pt samples for (a) the continuous film, (b) rounded NPs of about 10 nm diameter and (c) pyramidal NPs of about 6 nm diameter, all in 10 mbar CO at 425 K. For the thin Pt film, a characteristic peak of on‐top CO was observed at 2089 cm^−1^ in ppp, matching well the 2090 cm^−1^ on Pt(111) under these conditions^[41]^ (for details on fitting see the Supporting Information). As the surface roughness increases (i.e., the average Pt coordination number decreases), the on‐top peak shifts via 2086 cm^−1^ to 2057 cm^−1^. The ssp spectra mirror this trend, despite the slightly different phase. For the pyramidal 6 nm Pt particles, there is also a small peak at 2086 cm^−1^ in ssp, pointing to a few (111)‐like patches. Apart from the resonance position, the *I*_ssp_/*I*_ppp_ ratios are informative. The intensity ratios derived from the fitted peaks are collected in Table 1, together with the single crystal reference data.[^13^, ^14^, ^15^] Smooth films have no or few inclined (side) facets and also a low step/terrace sites ratio. Accordingly, SFG spectra of the thin Pt film show a strong ppp signal, but a very weak ssp signal. For the curved 10 nm Pt particles *I*_ssp_/*I*_ppp_ is highest (0.4), while it is smaller (0.3) for the pyramidal 6 nm Pt particles, due to their inclined, but less curved, surfaces.

**Figure 3:**
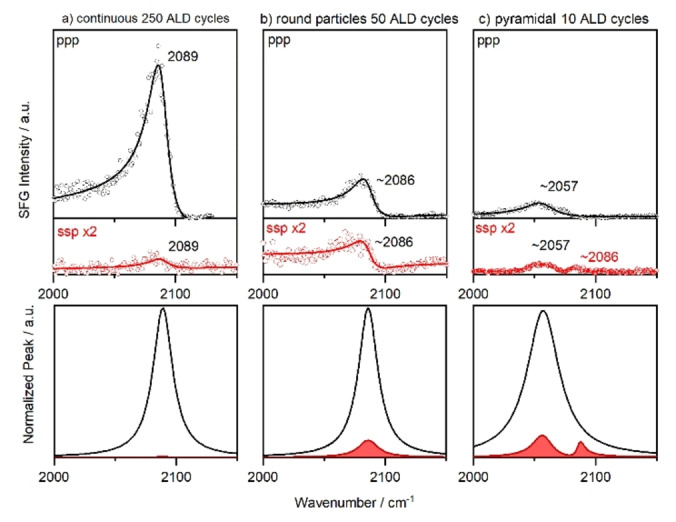
Polarization‐dependent SFG spectra of on‐top CO on different ALD‐grown Pt/ZrO_2_ samples. Measurements were performed in 10 mbar of CO at 425 K. The black spectra represent the ppp polarization combination, gradually shifting to lower wavenumber for smaller and rougher Pt nanoparticles. The red spectra represent the ssp polarization combination, which is enlarged by a factor of two with respect to the ppp spectra. SSP spectra are comparably stronger for curved particle morphology. The bottom row shows the normalized spectral fits of the resonant signal without the non‐resonant background.

**Table 1:** *I*_ssp_/*I*_ppp_ ratio (±10 %) for on‐top bonded CO on different Pt/ZrO_2_ samples and bridge bonded CO on different Pd/Al_2_O_3_ samples.

Pt on‐top CO *I*_ssp_/*I*_ppp_			
Single crystal	Film	10 nm NPs	6 nm NPs
0.04^[13]^	0.05	0.40	0.30
Pd bridge CO *I*_ssp_/*I*_ppp_			
Single crystal	6.1 nm NPs facetted	5.9 nm NPs rough	3.6 nm NPs rough
0.02^[13]^ sim. 0.10[^14^, ^15^] exp.	0.20	0.30	0.49

A previous polarization‐dependent study on Pt/SiO_2_ reported a significant enhancement of SFG intensities for both polarizations on 40 nm polycrystalline particles due to plasmon resonances.^[42]^ In our study we did not observe such an effect, which is likely due to the smaller overall size and different particle shape/structure.

For PVD‐grown Pd/Al_2_O_3_ model catalysts, SFG spectra of CO adsorbed on the Pd particles were obtained in UHV at 200 K, after saturating the surface with CO (cool‐down from room temperature in 10^−6^ mbar CO). The ppp and ssp polarization combinations for well‐facetted Pd particles of 6.1 nm mean diameter, as well as for rougher 5.9 and 3.6 nm Pd particles are shown in Figure 4. Compared to the Pt spectra discussed above, a strong asymmetric lineshape is visible in ppp, which is related to the NiAl substrate. As reported in detail in reference,^[43]^ it induces a much stronger non‐resonant background, likely related to an inter‐band transition in the substrate. Nevertheless, peak fitting still provides the resonance positions and normalized fits, excluding the non‐resonant background, as displayed in the bottom row of Figure 4. For all Pd NP sizes, ppp spectra predominantly showed bridge bonded CO (centered around 1981–1992 cm^−1^), adsorbed on particle edges and steps.[^7^, ^8^, ^29^, ^34^, ^35^, ^36^] This peak benefits from intensity transfer from the lower‐wavenumber bridged CO on (111) and (100) facets (shoulder around 1960 cm^−1^). The spectra also revealed a weaker on‐top CO centered around 2085–2096 cm^−1^. The ratio of *I*_ssp_/*I*_ppp_ for bridge bonded CO is given in Table 1, together with reference data from single crystal Pd(111), while the weak on‐top peaks were not included for Pd.

**Figure 4:**
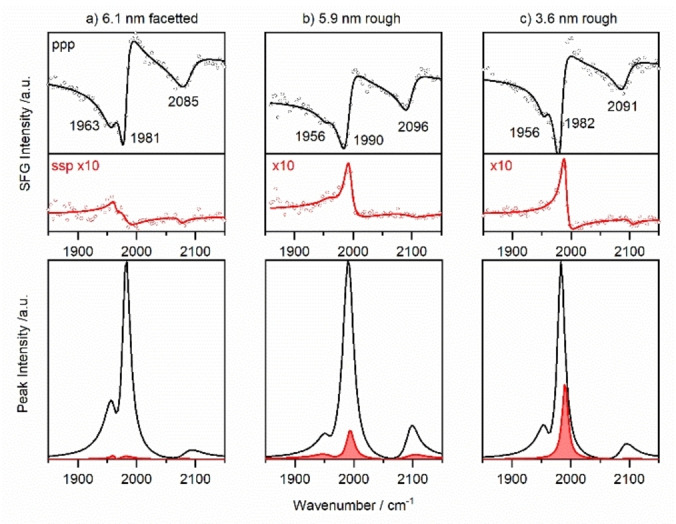
Polarization‐dependent SFG spectra of bridge bonded and on‐top CO on Pd/Al_2_O_3_ of various particle size and roughness. 6.1 nm particles were prepared at 300 K, 5.9 and 3.6 nm particles at 90 K. Measurements were performed in UHV at 200 K after saturating the surface with CO. The red spectra represent the ssp polarization combination, which is enlarged by a factor of ten with respect to the ppp spectra. SSP spectra are comparably stronger for curved particles. The bottom row shows the normalized spectral fits of the resonant signal without the non‐resonant background.

As described above, two distinct Pd particle shapes/morphologies were present. These shapes govern the *I*_ssp_/*I*_ppp_ ratio. The 6.1 nm Pd particles grown at 300 K are well‐facetted truncated cuboctahedra[^31^, ^32^, ^34^, ^35^] with a size/height aspect ratio of approximately 3. The “large” planar (111) top facets lead to a strong ppp signal, whereas the ssp signal of the smaller inclined side facets is weak. Accordingly, *I*_ssp_/*I*_ppp_ is quite small (0.2), approaching that of Pd(111) (0.1). The 5.9 nm Pd NPs prepared at 90 K have a similar mean diameter but have rougher and more pronounced side facets. As a result, the value of *I*_ssp_/*I*_ppp_ of 0.3 is higher. In comparison, the 3.6 nm Pd particles were also grown at 90 K and were thus rough. With their even smaller size, the *I*_ssp_/*I*_ppp_ intensity ratio is even larger (0.49).

In summary, polarization‐dependent SFG measurements were carried out for two model catalyst systems, consisting of different metal NPs (Pt vs. Pd), deposited by different methods (ALD vs. PVD), on different support materials (ZrO_2_ vs. Al_2_O_3_), with CO preferentially adsorbed on different binding sites (on‐top vs. bridge), at different CO pressures (10 mbar vs. UHV) and different temperatures (425 vs. 200 K). In both cases, in addition to the typically evaluated peak positions and intensities, the polarization‐dependent SFG measurements yielded *I*_ssp_/*I*_ppp_ ratios that reflect the particle morphology/surface curvature, in line with microscopic characterization. Based on this agreement, polarization‐dependent SFG spectroscopy can be applied for in situ characterization of particle morphology and especially changes thereof, even though SFG is usually not used for shape characterization. Note that this morphology evaluation is different from that of surface roughness, which is directly evident from shifts in the CO resonance position. Especially as an in situ spectroscopic technique, polarization‐dependent SFG allows observing changes upon treatments or during catalytic reactions (faceting, roughening, sintering etc.), while at the same time monitoring the reaction adsorbates/intermediates. This holds true for reactions that either involve CO or which are not affected by adsorbed CO. Herein, steady‐state spectra were acquired, but shorter acquisition times could be obtained by limiting the spectral range, automatic turning of polarizers or even broadband SFG. This presented approach may thus be utilized to characterize the morphology of model catalyst NPs during preparation, pretreatment and catalytic reactions.

## Conflict of interest

The authors declare no conflict of interest.

## Supporting information

As a service to our authors and readers, this journal provides supporting information supplied by the authors. Such materials are peer reviewed and may be re‐organized for online delivery, but are not copy‐edited or typeset. Technical support issues arising from supporting information (other than missing files) should be addressed to the authors.

Supporting Information

## Data Availability

The data that support the findings of this study are available from the corresponding author upon reasonable request.
